# Supplementary material for: A Humanized Yeast Phenomic Model of Deoxycytidine Kinase to Predict Genetic Buffering of Nucleoside Analog Cytotoxicity
Source: Genes (Basel). 2019 Sep 30;10(10):770. doi: 10.3390/genes10100770 (PMC6826991; doi:10.3390/genes10100770)
Supplement: Supplementary file 1 [file genes-10-00770-s001.zip › Additional_Files/Additional_File7_GOTermHeatmaps.zip!/Additional_File7_GOTermHeatmaps/maturation_of_LSU-rRNA_from_tricistronic_rRNA_transcript_(SSU-rRNA,_5.8S_rRNA,_LSU-rRNA).pdf]

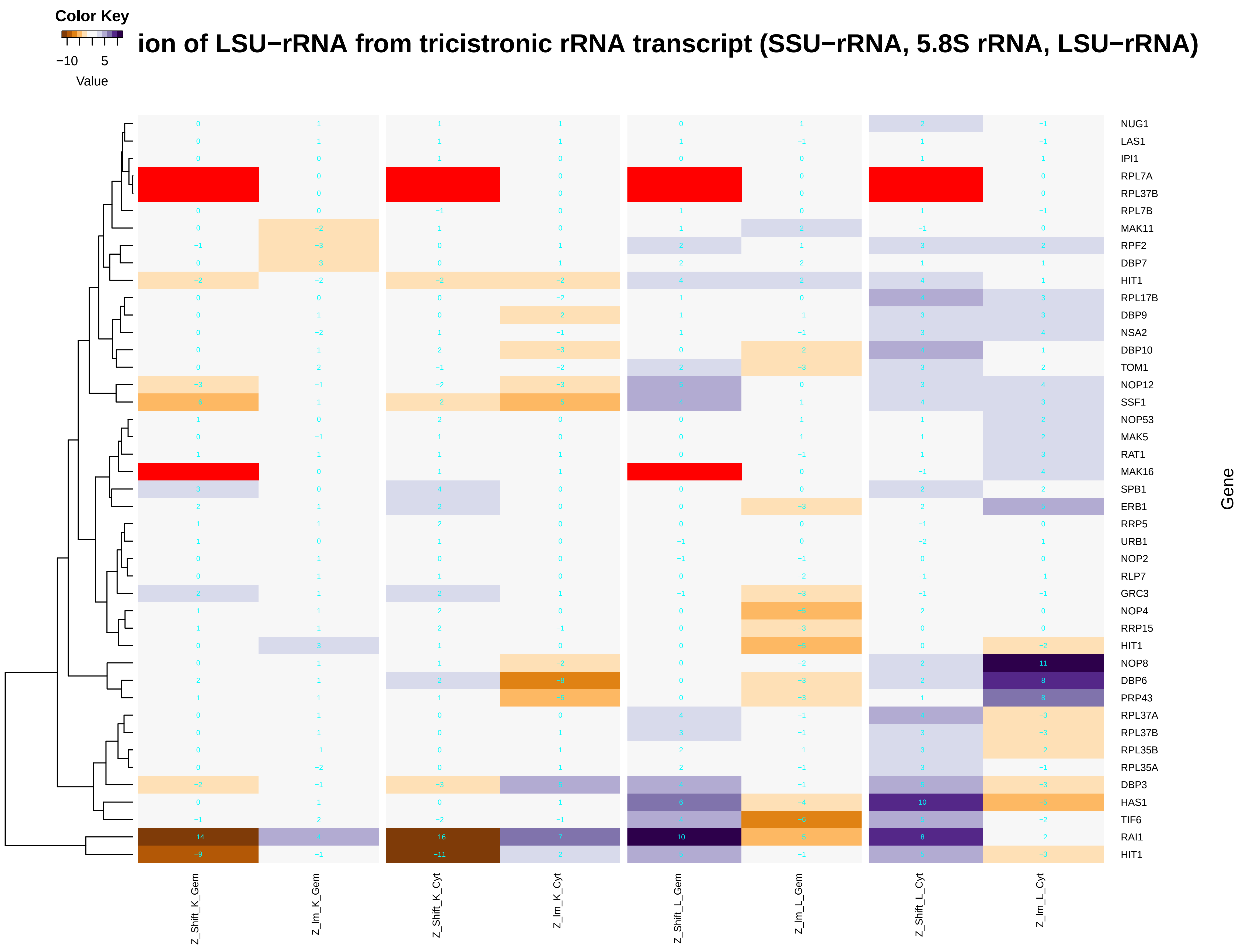
Color Key
−10 5
Value
ion of LSU−rRNA from tricistronic rRNA transcript (SSU−rRNA, 5.8S rRNA, LSU−rRNA)
NUG1
LAS1
IPI1
RPL7A
RPL37B
RPL7B
MAK11
RPF2
DBP7
HIT1
RPL17B
DBP9
NSA2
DBP10
TOM1
NOP12
SSF1
NOP53
MAK5
RAT1
MAK16
SPB1
ERB1
RRP5
URB1
NOP2
RLP7
GRC3
NOP4
RRP15
HIT1
NOP8
DBP6
PRP43
RPL37A
RPL37B
RPL35B
RPL35A
DBP3
HAS1
TIF6
RAI1
HIT1
Gene
Z_Shift_K_Gem
Z_Im_K_Gem
Z_Shift_K_Cyt
Z_Im_K_Cyt
Z_Shift_L_Gem
Z_Im_L_Gem
Z_Shift_L_Cyt
Z_Im_L_Cyt
